# Bilateral uveitis and macular edema induced by Nivolumab: a case report

**DOI:** 10.1186/s12886-017-0611-3

**Published:** 2017-12-01

**Authors:** Claire Theillac, Morgane Straub, Anne-Laure Breton, Luc Thomas, Stéphane Dalle

**Affiliations:** 1 Department of Dermatology, Service de dermatologie, ImmuCare, Centre Hospitalier Lyon-Sud, Hospices Civils de Lyon, 165 chemin du grand revoyet, Pierre Bénite Cedex, France; 2 Université Claude Bernard Lyon 1, Lyon, France; 3 Department of Ophthalmology, Centre Hospitalier Lyon-Sud, Hospices Civils de Lyon, Pierre Bénite Cedex, France; 4 Cancer Research Center of Lyon, Lyon, France

**Keywords:** Melanoma, Immunotherapy, Side-effect, Uveitis, Macular edema

## Abstract

**Background:**

Nivolumab is a fully human antibody which is routinely used at first therapy for metastatic melanoma. Usually, side effects are immune-related adverse events. We report a case of a man who developed bilateral anterior uveitis and macular serous retinal detachment during nivolumab treatment for metastatic melanoma.

**Case presentation:**

A man on nivolumab treatment for a leg melanoma with duodenal and lymph nodes metastases developed a sudden bilateral visual acuity impairment and bilateral non-painfull redness eyes several days after the third infusion. The clinical examination showed a significant decreased of the visual acuity. Slit lamp examination revealed the presence of bilateral granulomatous keratic precipitates, anterior chamber cells +++, bilateral synechiae, bilateral papilledema and macular edema associated with serous retinal detachment in the left eye. The anti-Programmed cells Death-1 was stopped and a topical corticosteroid treatment was administrated. After 8 days of topical corticosteroid treatment visual acuity was worsening with similar optical coherence tomography examination. An oral corticosteroid treatment was started. Evolution was favorable with a decrease of ocular inflammation and a complete visual acuity recovery after 3 weeks. Nivolumab was re-initiated.

**Conclusions:**

This is the second clinical report of bilateral anterior uveitis associated with macular serous retinal detachment related to anti-PD-1 treatment, and the first with nivolumab. Cases of uveitis were reported several times. Although rare, ophthalmologic manifestations that are rapidly recognized and adequately managed can be treated.

## Background

Nivolumab is a fully human IgG4 monoclonal antibody directed against the programmed cell death 1 (PD-1) receptor, which blocks inhibitory T-cell checkpoints. Nivolumab has been approvedas a first-line treatment for metastatic melanomasand is routinely used in clinical practice [1, 2]. Nivolumab has the same efficacy and safety outcomes for patients with wild-type or mutant, regardless of any previous treatment with BRAF inhibitors or with ipilimumab [3]. BRAF is a mutation of a human gene encoding a serine/threonine-specific protein kinase called B-Raf in MAP kinase pathway found in approximately 50% of multi metastatic melanomas. Clinical trials have proven the efficiency of these “checkpoint inhibitors” in treating metastatic melanomas, the risk of immune-related adverse events (irAEs) in practice is becoming increasingly known. IrAEs can affect all organs including the skin, the liver, and the digestive system [4]. The incidence of ophthalmologic side effects in patients treated by blocking PD-1/PD-L1 has so far not been reported. We describe a case of bilateral uveitis with anterior and posterior lesions in a patient with metastatic melanoma treated by nivolumab immunotherapy, an anti-PD1 drug.

## Case presentation

**Fig. 1:**
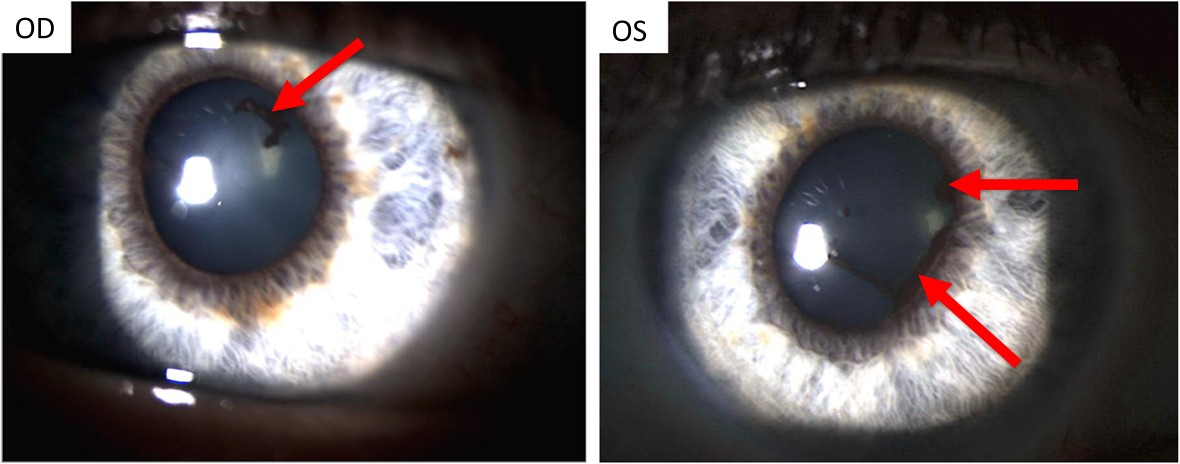
Bilateral slit lamp examination: OD and OS: posterior synechiae (red arrows)

**Fig. 2:**
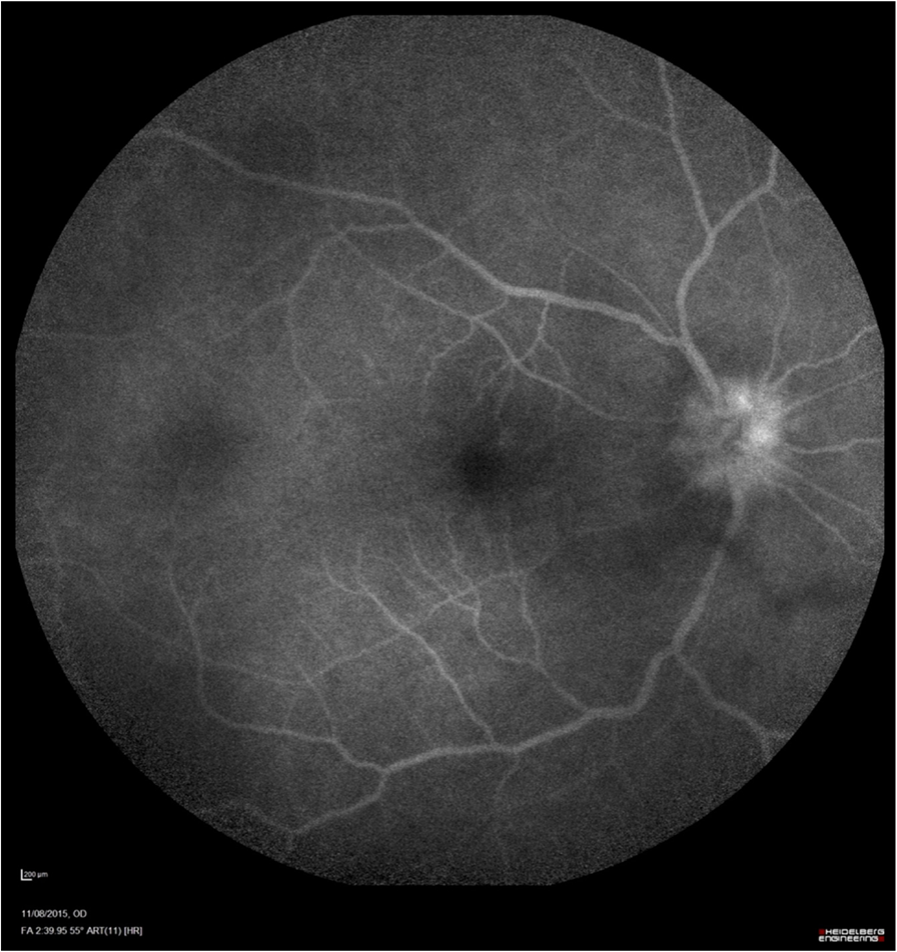
Right eye fluorescein angiography with papillitis (early stage 2:39 min)

**Fig. 3:**
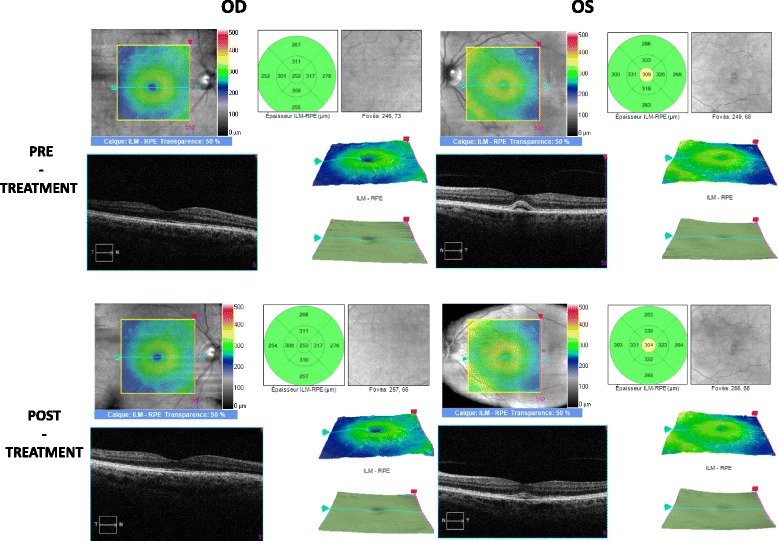
Bilateral optical coherence tomography: OD: normal foveolar profile before and after treatment, OS: retrofoveal subretinal detachment before and after treatment

A 55-year-old Caucasian patient was treated with nivolumab as a second line therapy for metastatic melanoma, affecting the lymph nodes and duodenum, harboring a BRAF V600E mutation. The primary melanoma located on the left leg had been removed fifteen years earlier. A relapse was diagnosed following a paraneoplasic syndrome including vitiligo and severe anemia consecutive to gut bleeding. According to metastatic melanoma french recommendations, the patient received vemurafenib (anti-BRAF) as a first line therapy without developing any significant side effects. The 3-month tumoral evaluation by CT-scan revealed a progressive disease, according to the Response Evaluation Criteria In Solid Tumors (RECIST). A second line of treatment with nivolumab (3 mg/kg every 2 weeks) was then initiated. Although no adverse events were observed after the first two infusions of the anti-PD1 antibody, the patient complained of sudden bilateral visual acuity impairment several days after the third infusion. The ophthalmologic evaluation showed a significant decrease of the visual acuity (20/20 OD, 20/40OS) associated with a non-painful redness in both eyes. Slit lamp examination revealed the presence of bilateral granulomatous keratic precipitates, anterior chamber cells +++,bilateral anterior and posterior synechiae, predominant in the left eye and some pigmentary deposits on the anterior lens capsule [Fig. 1].The ocular fundus interpretation was limited because of the anterior segment inflammation. Bilateral papilledema (papillitis) was confirmed by fluorescein angiography [Fig. 2] and indocyanin green (no vasculitis, no retinal foci). Optical coherence tomography (OCT) showed a light macular edema associated with a subfoveal serous retinal detachment on left eye. The ophthalmic diagnosis was bilateral granulomatous uveitis and unilateral posterior retinal serous detachment (OS). Common causes of bilateral granulomatous uveitis like sarcoidosis, syphilis, and tuberculosis were first ruled out, and our final diagnosis was an anti-PD1 induced uveitis and retinopathy. Local treatment with a topic corticosteroid eye drops (sodium phosphate dexamethasone 0.1%) was initiated. While a decrease of conjunctival redness and pain was observed on day 8, the second ophthalmologic examination revealed that the visual acuity was still declining. The visual acuity scored 20/20 for the right eye and 20/50 for the left eye with a persistent bilateral uveitis (uveitis grade 2 in Common Terminology Criteria for Adverse Event (CTCAE)). Macular edema and serous retinal detachment in the left eye was similar on OCT (retinopathy grade 2 in CTCAE) [Fig. 3]. An oral corticosteroid treatment (1 mg/kg), prednisone, was initiated after a discussion between ophthalmologist and dermato-oncologist teams.

The examination conducted on day 20 revealed an improvement of visual acuity (20/20 for both eyes), reduction of retrodescemetic precipitates, disappearance of the Tyndall effect, persistent synechiae, and decrease of serous retinal detachment on left eye. Administration of oral systemic corticosteroids and corticosteroid eye drops were continued for 2 weeks before doses were progressively reduced.

We observed a successful outcome after oral corticosteroid treatment: a decrease of ocular inflammation and a complete visual acuity recovery after 1 month. Treatment with nivolumab was re-initiated and corticosteroids were gradually decreased. No relapse of bilateral uveitis occurred but corticosteroids were not completely stopped on Nivolumab, we had to change therapy because of tumoral increase during corticosteroids decreasing (3 months and 5 infusions later).

## Discussion

In the era of anticancer immunotherapies it is crucial to distinguish drug-related effects with potential auto-immune related causes and common toxic side effects. Anti-CTLA-4 and anti-PD1 are both “immune check point inhibitors” acting differently (two different pathways). Anti-CTLA-4 works by promoting T cell responses that are largely non specific for tumors antigens, whereas the most important principle of anti-PD1 therapy is its localized effect (PD-L1 expression is mostly in tumor microenvironment). Because anti-PD1 therapy selectively modulates inflammatory T cells response at the tumor sites, it has low immune complication. The FDA approved two anti-PD1 to treat human cancer, one from Bristol-Myers Squibb (nivolumab) and another from Merck (pembrolizumab) [5]. Nivolumab is the most cost effective therapeutic strategy in France.

The most commonly observed immune related events following treatment with anti-PD1 concern skin (pruritus, rash), bowel, lung [6, 7], liver, hypophysis, and thyroid. So far, only clinical trial data are available for treatments with nivolumab, and they generally show a lower frequency of adverse events when compared to anti-cytotoxic T-lymphocyte associated antigen 4 (anti-CTLA-4) [8]. In the study by Suzanne L. Topalian et al. (2012) [6], grade 3 or 4 drug-related adverse events occurred in up to 14% of patients, while serious drug-related adverse events occurred in 11% of patients. Hepatic and gastrointestinal adverse events were reversible in all cases after treatment discontinuation and use of systemic steroids. Endocrine disorders were managed with hormone supplementation.

Ophthalmologic side effects have scarcely been reported in relation to anticancer immunotherapies. Using ipilimumab (anti-CTLA-4), the eyes were affected by immune related adverse events in less than 1% of patients [8]. These events were generally resolved within a week. While treatment with corticosteroid eye drops are often considered first, systemic corticosteroids are strongly recommended in the more severe cases, such as uveitis, iritis, or episcleritis. Uveitis associated with anti-CTLA-4 is described several times and ocular side effects affect about 1.3% of ipilimumab treated patients [9, 10]. The main ophthalmic side effect induced by pembrolizumab (anti PD-1) is uveitis [10–16]. This is the first case of macular edema reported with nivolumab and the fourth uveitis [17–19].

When adequately managed and after a multidisciplinary discussion, the anti-PD-1 therapy may be re-introduced, with a corticosteroid treatment progressive decrease and eventually stop with low risk of a relapse of the immune related adverse events in most cases. Several studies indicate that increased autoimmunity might be associated with better tumor control even though this notion was not supported in recent case series [8, 9]. Immune side effects appear to be related with increased tumoral activity in our case.

## Conclusion

We report a symptomatic bilateral anterior uveitis (grade 2) with serous retinal detachment in a patient treated with nivolumab, an anti-PD-1 antibody approved for melanoma and lung cancer therapies. These ocular lesions resulted from an immune related reaction induced by nivolumab. Although rare, ophthalmologic manifestations that are rapidly recognized and adequately managed can be cured. In our case, the uveitis and serous retinal detachment receded with oral and topical corticosteroid treatment and the discontinuation of nivolumab during 2 months.

## Abbreviations

BRAF mutation: Mutation of a human gene encode a serine/threonine-specific protein kinase called B-Raf (MAP kinase pathway)
CTCAE: Common Terminology Criteria for Adverse Event
CTLA-4: Cytotoxic T-lymphocyte associated antigen 4
irAEs: Immune-related adverse events
OCT: Optical Coherence Tomography
OD: Right eye
OS: Left eye
PD-1: Programmed-cell Death 1
RECIST: Response Evaluation Criteria In Solid Tumors
